# The Pulsing Paradox: Successful Steroid Therapy in Infection-Related Glomerulonephritis

**DOI:** 10.7759/cureus.64769

**Published:** 2024-07-17

**Authors:** Ananya Anantharaman, Viswanathan Pandurangan, Devasena Srinivasan, Divya Joyce, Subalakshmi Balasubramanian

**Affiliations:** 1 General Medicine, Sri Ramachandra Institute of Higher Education and Research, Chennai, IND; 2 Pathology, Sri Ramachandra Institute of Higher Education and Research, Chennai, IND

**Keywords:** infection-related glomerulonephritis, acute nephritic syndrome, systemic steroids, rapidly progressive glomerulonephritis (rpgn), staphylococcal cellulitis

## Abstract

The patterns of infection-related glomerulonephritis (IRGN) are rapidly changing in terms of age at presentation and sources of infection. The existing literature on the use of steroids in IRGN is inconsistent. A diabetic male in his sixties presented with features of anasarca, bilateral flank pain, and acute pulmonary edema. He had a non-healing ulcer over his right leg, with pus culture showing growth of methicillin-resistant *Staphylococcus aureus* (MRSA). Computed tomography (CT) of the kidneys, ureter, and bladder (KUB) showed features of bilateral pyelonephritis. The patient went on to develop acute renal failure and eventually required hemodialysis. A renal biopsy was performed, and features of IRGN with crescents were noted. Considering the presence of crescents in renal biopsy, a trial of steroids was given under antibiotic cover, which resulted in a near-complete resolution of renal failure.

## Introduction

The term infection-related glomerulonephritis (IRGN) has replaced the term post-infectious glomerulonephritis since many patients still have active infections at the time of renal injury [1]. It is proposed that IRGN is secondary to molecular mimicry or antigen-antibody complex deposition. The epicenter is often an infection of heterogeneous etiologies. The common sites of infection are the skin and upper respiratory tract, often secondary to *Staphylococcus* and *Streptococcus* species [2]. Recently, there has been an increasing association between gram-negative organisms and deep-seated visceral infections [3]. Adults with long-standing diabetes, alcoholism, chronic liver disease, or chronic kidney disease tend to experience worse outcomes, such as nephrotic-range proteinuria and dialysis dependency [3]. In fact, IRGN is the second most prevalent non-diabetic kidney disease among elderly diabetics [4]. There is very little data on the outcomes or treatment options in IRGN patients with underlying diabetic kidney disease. When patients develop worsening renal failure despite receiving appropriate antibiotic therapy for source control, there is a cause for significant suspicion and evaluation of IRGN [5].

Despite immunological dysfunction being at the core of its pathogenesis, IRGN has not shown promising results with immunosuppressive therapy. Randomized control trials pertaining to the use of steroids in IRGN are scant, and studies that are available bear conflicting results [6-8]. More study in this area is crucial, given that a significant portion of IRGN patients end in death or end-stage renal disease (ESRD).

## Case presentation

An elderly diabetic and hypertensive male in his sixties first noticed a non-healing ulcer over his left ankle two months ago associated with purulent discharge, pain, and swelling following an injury. Three weeks prior to the current presentation, he developed diffuse abdominal distension, facial puffiness, bilateral leg swelling extending from ankles to knees, and breathlessness (progressing from New York Heart Association Grade II to Grade IV). He also developed haematuria and decreased urine output over the last 10 days. He is poorly compliant with medications. His personal habits included alcohol consumption of 180 ml/6.08 fluid ounces of hard liquor (roughly 72 grams) per day for the last 20 years. He had no history of consumption of non-steroidal anti-inflammatory drugs, alternative medication intake, fever, or dysuria.

On examination, the patient was conscious, oriented, and afebrile, with a pulse rate of 78 beats per minute and a blood pressure of 180/100 mmHg. Features of anasarca were noted, along with elevated jugular venous pressure (12 cmH_2_O above the right atrium) and bilateral pitting type of pedal edema. Upon lung auscultation, the patient was found to have bilateral crepitations. An abdomen examination showed a distended abdomen with free fluid and bilateral renal angle tenderness. An ulcer of 3x3x2 cm in size was seen 4 cm above the lateral malleolus of the left leg, with surrounding erythema, warmth, and tenderness. Admission investigations are shown in Table 1.

**Table 1:** Admission laboratory parameters WBC - white blood cells; HbA1c - glycated hemoglobin; SGOT - serum glutamate-oxaloacetate aminotransferase; SGPT - serum glutamate-pyruvate aminotransferase; BUN - blood urea nitrogen

Parameter	Patient value	Reference range
Hemoglobin (g/dL)	12.3	12–17
Total WBC count (x10^3^/mm^3^)	8.73	4–11
Platelets (x10^3^/mm^3^)	197	150–450
HbA1c (%)	8.3	4–6
Microvascular complications of diabetes	No evidence of diabetic retinopathy	Not applicable
Total bilirubin (mg/dL)	0.5	0.1–1.2
SGOT (U/L)	24	0-35
SGPT (U/L)	14	0–41
Serum total protein (g/dL)	5.9	6.4–8.2
Serum albumin (g/dL)	2.9	3.2–4.8
BUN (mg/dL)	14	7–18
Creatinine (mg/dL)	1.0	0.6-1.3
Sodium (mmol/L)	138	134-144
Potassium (mmol/L)	4.1	3.5-5
Total cholesterol (mg/dL)	200	<200

An electrocardiogram (ECG) showed normal sinus rhythm. A 2D transthoracic echocardiogram showed no regional wall motion abnormalities, an ejection fraction of 60%, and no significant diastolic dysfunction. Troponin-I was <0.01 ng/mL (normal <0.02 ng/mL), and B-type natriuretic peptide was 120 pg/mL (normal <100 pg/mL). Ultrasound of the abdomen showed normal liver span and echotexture with normal portal vein diameter and no evidence of any collaterals. Renal cause for anasarca was considered and thus evaluated accordingly (Table 2).

**Table 2:** Evaluation of renal causes of anasarca with imaging and urine routine analysis CT-KUB - computed tomography of kidneys, ureters, and bladder

Parameter	Patient value	Reference range
CT-KUB	Features of bilateral pyelonephritis; no evidence of obstruction	Not applicable
Urine routine: proteins	3+ on dipstick	Negative
Urine routine: glucose	4+ on dipstick	Negative
Urine routine: red blood cells	151 red blood cells per high-power field	< 5 cells
Urine Protein: creatinine ratio	9.50	<0.2
Urine spot protein	408 mg/dl	<150 mg/dl
Urine spot creatinine	43 mg/dl	37– 250 mg/dl

Pus aspirate taken from the wound site grew methicillin-resistant *Staphylococcus aureus* (MRSA). Urine and blood culture yielded no growth. Bilateral lower limb arterial and venous doppler showed no evidence of deep vein or arterial thrombus and confirmed the presence of cellulitis underneath the ulcer. A clinical diagnosis of soft tissue sepsis with bilateral pyelonephritis and anasarca secondary to diabetic nephropathy was made.

The patient was started on piperacillin-tazobactam 4.5 g intravenously thrice daily for linezolid 600 milligrams per oral twice daily for the leg ulcer. Diuretics and anti-hypertensives were initiated. He additionally underwent regular wound debridement as a part of infection source control. Glycaemic control was achieved during the course of the stay.

**Figure 1:**
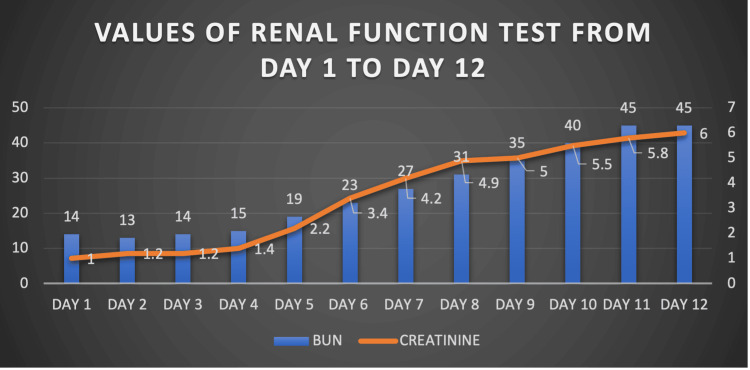
The values of renal function test from day one to day 12 BUN - blood urea nitrogen

The patient initially symptomatically improved on intravenous antibiotics. However, he went on to develop worsening renal parameters (Figure 1) along with gross haematuria and oliguria. Features of pulmonary edema worsened, requiring non-invasive ventilation (NIV), and blood pressure remained persistently elevated despite optimal therapy. The patient was initiated on hemodialysis, the requirement of which was noted every alternate day. Repeat renal imaging revealed resolving pyelonephritis with no evidence of obstructive uropathy. A urine culture was repeated prior to renal biopsy, which yielded pan-resistant *Escherichia coli*. Ceftazidime-avibactam (1.25 g intravenously eighth hourly) and aztreonam (1 g intravenously once a day) were initiated with renal adjustment.

The suspicion of IRGN was then raised in view of the temporal relationship of decline in renal parameters, haematuria, proteinuria, poor blood pressure control, and pulmonary edema with the onset of infection. Other possible causes for rapidly progressive glomerulonephritis (RPGN) were also evaluated (Table 3).

**Table 3:** Evaluation for other causes of RPGN ESR - erythrocyte sedimentation rate; CRP - C-reactive protein; anti-HBs - anti-hepatitis B surface antigen; anti-HCV - anti-hepatitis C virus; HIV - human immunodeficiency virus; ASO - anti-streptolysin O; ANA - anti-nuclear autoantibodies; anti-dsDNA - anti-double-stranded DNA; ANCA - anti-neutrophil cytoplasmic autoantibodies; RF - rheumatoid factor; anti-CCP - anti-cyclic citrullinated protein

Parameter	Patient value	Reference range
ESR (mm/hr)	94	<32.5 (age and gender adjusted)
CRP (mg/dL)	6.1	<1.3 (age and gender adjusted)
Serum C3	45	90–180
Serum C4	19	10–40
Viral markers (anti-HBs, anti-HCV, HIV I/II p24)	Negative	Negative
ASO titre (IU/mL)	259.0 international units/milliliter (IU/ml)	<116
ANA by IF, anti-dsDNA titre, ANCA, RF, Anti-CCP	Negative	Negative

**Figure 2:**
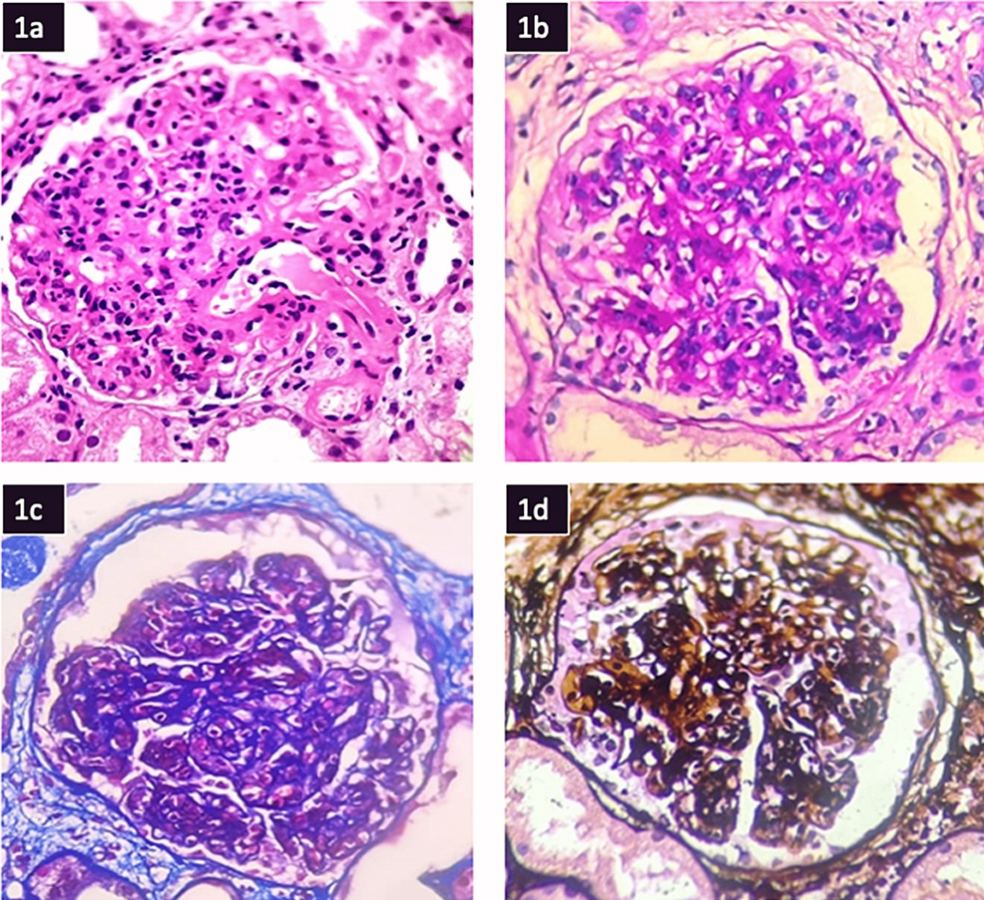
a) Hematoxylin & Eosin stain showing endocapillary and mesangial hypercellularity and lobular accentuation of the glomerulus along with hyalinosis of the afferent arteriole (x400); b) Periodic acid Schiff stain showing mesangial matrix expansion along with endocapillary hypercellularity X400. c) Masson trichrome stain (x400). d) silver stain (x400) Twenty-two glomeruli were observed, showing an exudative pattern of injury with neutrophilic infiltration, causing mesangial and endocapillary hypercellularity with glomerular basement membrane thickening and focal segmental lobular accentuation. Cellular crescents were seen, and one complete and two partials were noted. Twenty percent of tubules were noted to be atrophied. Tubular injury in the form of loss of brush border, regenerative atypia in the form of nucleomegaly, and prominent nucleoli are seen. Hyaline and RBC casts are present. Features of edema and scattered lymphocytic infiltrates were seen. Interstitial fibrosis of 20% was noted. Endothelial cell prominence, intimal thickening with hyalinosis, fibrin deposition, and medial wall thickening were present. A luminal narrowing of 30% was seen.

**Figure 3:**
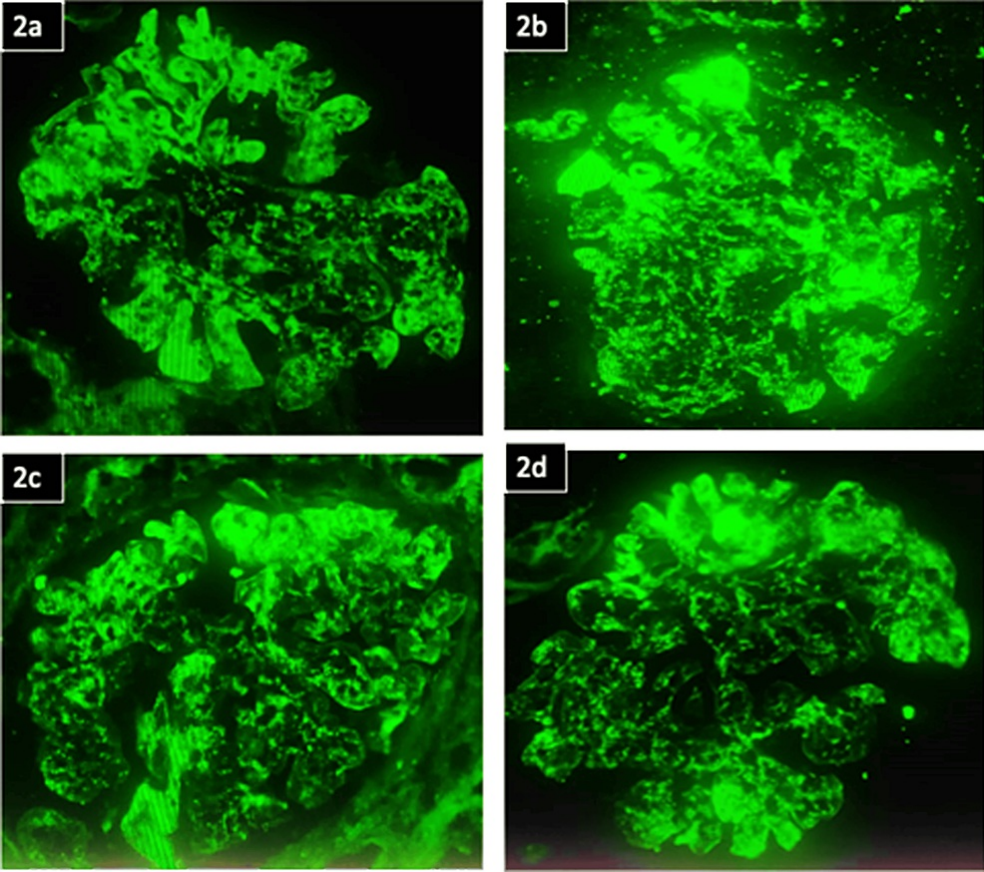
Immunofluorescence of renal biopsy specimen Granular capillary wall and mesangial 3+ positivity for IgG, C3, Kappa, and Lambda were noted. All other conjugates were negative. C3 vessel wall positivity noted. The green stain represents the areas staining positive for IgG, C3, Kappa, and Lambda, on immunofluorescence microscopy.

**Figure 4:**
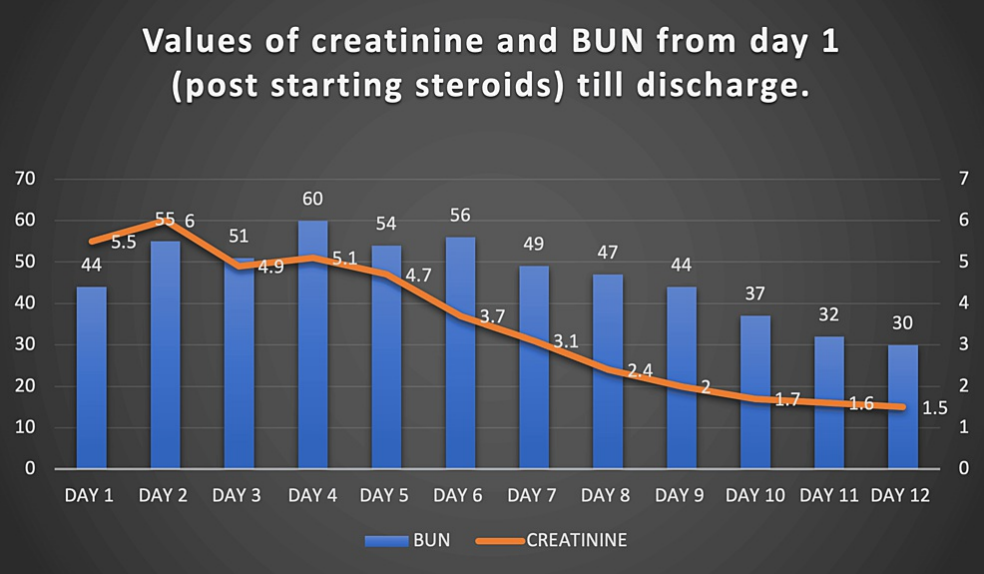
The values of renal function from day one of starting steroids till discharge BUN - blood urea nitrogen

Renal biopsy showed features of IRGN with crescents against a background of diabetic nephropathy (Figures 2, 3). Following the biopsy report, he was started on steroids (prednisolone 40 mg per day) under the cover of ceftazidime-avibactam and aztreonam. He proceeded to show good clinical improvement. Serial monitoring of renal function tests showed a decreasing trend (Figure 4). Hematuria settled, and urine output improved.

He was slowly tapered off all ventilatory supports, and no further hemodialysis requirement was noted. The patient was hospitalized for a total of 30 days, with a complete resolution of pulmonary edema, hematuria, and oliguria; at discharge, a near-normal creatinine (1.3 mg/dL) was noted. Steroids were tapered on an outpatient basis. On follow-up in the clinic two weeks later, the patient had normal renal parameters and was educated on optimal glycaemic control, alcohol abstinence, and necessary lifestyle modifications.

## Discussion

This case report intends to emphasize that, in the context of established infection, systemic or localized, persistent derangement of renal function raises the possibility of IRGN and warrants evaluation for the same. This case report also aims to discuss the role of steroids in IRGN when combined with antibiotics and source control techniques.

The major risk factors of IRGN in the elderly include diabetes, alcoholism, chronic liver disease, chronic kidney disease, and other immunocompromised states [3]. Kurien et al. showed that IRGN is the most common finding in renal biopsy in the Indian geriatric population [9]. Existing literature on the correlation of alcohol to IRGN suggests that patients with chronic alcoholism tend to have worse outcomes [10,11]. The association between alcohol and diabetes is attributed to poor oral hygiene, possible dysbiosis, and underlying immunological compromise [3,4]. While it has been demonstrated that IRGN can be linked to deep-seated visceral infections, data suggesting a link between IRGN and pyelonephritis is lacking.

The diagnosis of IRGN can be made based on the presence of any three out of the following five parameters: 1) definitive evidence of infection at the time of or just preceding the glomerulonephritis, 2) hypocomplementemia, 3) exudative glomerulonephritis, 4) C3 dominant immunofluorescence, and 5) subepithelial hump-shaped deposits on electron microscopy [12]. The most common histopathological pattern of renal injury in IRGN is an exudative type of glomerulonephritis with diffuse endocapillary proliferation and neutrophilic infiltration. Other patterns seen are mesangial and focal proliferative typess of glomerulonephritis. The least common type of all is shunt nephritis. Shunt nephritis is a membranoproliferative type of glomerulonephritis occurring in cases of *Staphylococcus epidermis* infections in patients with vascular shunts [2]. The key differentials to IRGN include lupus nephritis, immunoglobulin A-associated vasculitis-related glomerulonephritis, C3 glomerulopathy, cryoglobulinaemic glomerulonephritis and, in the setting of endocarditis, anti-neutrophil cytoplasmic antibody-associated RPGN [13]. Differentials are noted in Table 4.

**Table 4:** A comparison of the close mimickers of IRGN Based on studies by Nasr et al. [14], and Khalighi et al. [15] IRGN - infection-related glomerulonephritis; ASO - antistreptolysin O

Entity	Serum C4	Serum C3	Other features
IRGN	Normal	Low	Positive bacterial cultures, raised ASO-titres, sub-epithelial humps, exudative glomerular injuries
Lupus nephritis	Low	Low	
C3 glomerulopathy	Normal	Low	Differentiated from IRGN based on: consistently low complement values more than eight weeks after presentation; persistent microscopic hematuria, hypoproteinemia; significant proteinuria for more than a year post presentation; repeat biopsy showing C3 predominance on immunofluorescence
Cryoglobulinaemic glomerulonephritis	Low	Normal	
IgA nephropathy	No data	No data	Close mimicker of IgA-associated IRGN, which has culture proven *Staphylococcus* infection, exudative glomerular infiltration, C3 dominance over IgA on immunofluorescence and sub-epithelial humps on electron microscopy and predilection for diabetic nephropathy
ANCA-associated RPGN	No data	No data	8% of IRGN elderly patients may have anti-MPO or anti PR3-ANCA positivity

The primary indication for biopsy in IRGN is to rule out other causes of rapidly progressive glomerulonephritis, a few of which could necessitate aggressive and rapid-onset immunosuppression. Biopsy may be avoided in those cases on IRGN, which resolve spontaneously with antibiotics along with established hypocomplementemia and infection occurring just prior to, or concurrently with renal injury.

IRGN in adults is more challenging to manage than in children for two reasons. IRGN in adults is often associated with an ongoing infection, the treatment of which becomes the cornerstone of management. This concurrent infection is also a contraindication to aggressive immunosuppression. IRGN also has worse outcomes compared to the near-total resolution in children. In addition to this, it also closely resembles the aforementioned differentials, resulting in a delay in optimal therapy. Complications like congestive cardiac failure, pulmonary edema, acute renal failure, and hypertensive or uremic encephalopathy may necessitate intensive care.

The management of IRGN is usually supportive and includes salt restriction in diet, anti-hypertensives, diuretics, and targeted antibiotic therapy for the complete eradication of the underlying infection. Usage of angiotensin-converting enzyme inhibitors may be warranted in case of nephrotic-range proteinuria.

Most literature describing the use of steroids in IRGN currently available is retrospective and anecdotal. The few prospective studies available do not show an explicit superiority benefit of steroids in IRGN. A recently performed prospective study failed to show any significant recovery from renal failure when treated with steroids [8]. However, this was an open-label, single-center trial performed on a small study group that did not attain the targeted sample size. A study conducted by Nagaba et al. attempted the use of steroids in patients with crescents on biopsy and completed eradication of the methicillin-resistant *Staphylococcus aureus* (MRSA) infection. However, the patients in the steroid arm of the trial had relapses of the disease and succumbed to sepsis. This study suffers from the limitation of a small study population as well [16]. Studies also show that the usage of steroids as stand-alone therapy does not bear promise, and antibiotics are an absolute necessity [6].

Other trials list indications for steroid therapy in cautious concert with antibiotics to provide better results, as opposed to the sole use of antibiotics. A retrospective study conducted by Baikunje et al. showed promising results when steroids were used in patients of age less than 50 with IRGN with crescents and nephrotic-range proteinuria [17]. When antibiotic therapy has failed to produce any improvement in renal function, crescents are established in more than 30% of the glomeruli observed on biopsy, and eradication of underlying infection is certain, then steroids may be attempted [18]. It is also recommended to continue the use of antibiotics even if infection has been controlled [19,20]. These studies have documented an improvement in renal function and reduction in dialysis dependency even when antibiotics alone have failed to demonstrate a response.

## Conclusions

IRGN is a complex entity involving the simultaneously-occurring duality of infection and immune dysregulation. It also brings along the morbid burden of end-stage renal disease. While antibiotics continue to take the center stage of management, when used with caution, steroids may be a useful treatment strategy. Further studies are required to demonstrate their benefit.
